# Malaria and Its Manifestations

**Published:** 1893-03

**Authors:** A. G. Clopton

**Affiliations:** Professor of Physiology and Hygiene in the Medical Department of the University of Texas


### DANIEL'S TEXAS MEDICAL JOURNAL.

ESTABLISHED JULY, 1885.

PUBLISHED MONTHLY.—SUBSCRIPTION $2.00 A YEAR.

Vol. VIII. AUSTIN, MARCH, 1893. No. 9.

### Original Contributions.

For Daniel's Texas Medical Journal.

### MALARIA AND ITS MANIFESTATIONS.

BY A. G. CLOPTON, M. D.,
Professor of Physiology and Hygiene in the Medical Department of the University of Texas.

[Read before the Galveston County Medical Society, January 5, 1893.]

WHEN notified that I was expected to prepare a paper for this meeting of the Galveston County Medical Association, I chose as my subject the mechanism of fever, but upon reflection, concluding that such a subject would be only of abstract interest to the members, abandoned it. The theme of my paper is: "Malaria and its Manifestations." I chose this subject because it is of practical importance and because it is replete with interest to every practising physician, and not because I have any original thoughts to present. You are as familiar with the subject, perhaps, as I am, and no doubt will join issue with me upon more than one point discussed. I invite your criticism upon any position assumed in this paper, but not upon my imperfect style or feeble argument—to these defects I plead guilty, thrice guilty. Light, more light is the all-felt want of every searcher after truth; and discussion often exposes error. There are many false facts in medicine, or at least theories accepted as facts, which will be proven errors. It has been so in the past, and will no doubt be so in the future. Exstinguished theories lie along the pathways of science like strangled snakes around the cradle of Hercules.

My paper will be suggestive rather than comprehensive, propounding questions rather than answering them. What is malaria? Bacteriologists have definitely solved this question, but its solution leaves many points of interest in as great mystery as before. Malarial manifestations are phenomenal in their variety and diversity. Malarial poison is a micro-organism, which, when introduced into the animal body, multiplies by segmentation or otherwise, and under favorable condition develops a specific fever. Are these microbes restricted to originating a well defined specific lever, or do their manifestations vary, as conditions and environments vary? Do they develop only intermittent and remittent fevers, or do they manifest their presence in protean forms, with or without fever? The older writers and physicians recognized manifold manifestations, and plausibly under their theory explained them. Authors and physicians, since the general acceptance of the bacterian theory, while restricting malarial manifestations somewhat, acknowledge different forms, and in their attempt to explain them, darken the mystery; they dovetail their explanations, but give no more light. If malaria is the result of a specific microbic poison, does it not defy a general pathological law, which governs all other microbic poisons, as far as we know? Are these microbes veritable pathological outlaws, defying regulation and fantastic in their manifestations? Other specific poisons develop specific diseases, *sui generis*, mild or malignant, simple or complicated, but definite and specific. Typhoid fever bacteria manifest their origin in the animal economy, if at all, by originating typhoid fever. There is but one typhoid fever; its group of symptoms may vary somewhat, but it is typical and its pathological lesions are always the same and never mask another disease. The exanthemata are never convertible; each virus develops a specific disease after its kind. Cholera, typhus fever, yellow fever, observe the same law. Does this general pathological law apply to the *plasmodium malariæ?* Does it not play tricks ever upon its specific fever, so much so that our nomenclature groups its manifestations under special names, intermittent and remittent,—and the former of these is never constant, quotidian, tertian, quartan? Do all these different types originate from the same micro-organism or are there as many different parasites as there are manifestations? Before the discovery of the *plasmodium malariæ* we endeavored to explain these differences by assuming that there were different malarial poisons, and these varied manifestations were the results of different conditions and environments. Thus we talked learnedly of paludal and dry malaria, of civic and country malaria, and many other kinds of malaria—all nonsense, we now know. Yet even now we attempt to explain these differences upon the same line of assumption. We now claim that there are different malarial parasites, each playing its own role upon the pathological stage. Thus Lavaran describes transparent, colorless, spheroidal bodies, delicate, tenuous bodies, crescentic bodies. He denies the plurality of these different bodies, believes they are polymorphous, but single, and their evolution is not the same.

Another distinguished investigator, Golgi, explains the different types of malarial fever by assuming that each type has its special parasite, having a period of development of two days for the tertian form, a quartan depending upon a parasite having a period of development of three days, and an intermittent fever, the type of which he does not specify, depending npou a parasite the significance of which has not been determined, and thus in his profound research he may go on *ad infinitum*, discovering a parasite for every manifestation. I would not criticise so able an investigator but must believe his theory scientific nonsense. I may be allowed to quote the language of Agrippa to Paul: "Too much learning has made this bacteriologist mad." What scientific straits are we driven to in explanation of the tricks of plasmodium?

But these malarial microbes are not only outside of a general pathological law in their phenomenal manifestations, but in other things as well. All other specific diseases, originating from specific microbes, except perhaps dengue and the so-called "grippe," of which we know next to nothing, give immunity more or less complete, from subsequent attacks. These little outlaws give no immunity but predispose to subsequent attacks. Again, they have no stage of incubation; there is a period of latency shorter or longer, but no period of incubation, as I understand the term. Two men susceptible to the malarial poison may sleep in the moist swamp, aud one develop intermittent or remittent fever in twelve hours or less, and the other in six months after exposure. Undoubted instances of malarial fever long after exposure have been reported. Prof. Brown reports the following facts: Twelve persons, in 1865, visited Barkum, an island of East Friedland, for the purpose of taking baths; two of these suffered from intermittent fever while on the island; nine of the others escaped until the following spring and summer, when they were seized with the fever (six and nine months after exposure). The plasmodium of malaria will remain latent, perhaps, for years, developing no fever or other manifestation as long as the person remains in a malarial district, but as soon as he leaves for the mountains or a non-malarial district, he will be seized with intermittent fever. Such cases have often come under my observation. Again, the effects of the malarial micro-organism are not always self-limited, as are those of typhoid or typhus fever or the exanthemata. These diseases have their severe and dangerous sequilæ but they are always limited in their duration. They never become chronic; intermittent fever may become chronic, establishing a malarial cachexia, lasting a life-time. Verily, these malarial parasites are pathological outlaws or they are governed in their manifestations by laws which have as yet not been revealed. They manifest their presence in protean forces. May not relapsing fever be one of its forms? If Lavaran and other bacteriologists are not in error it may be the product of one of the many forms of the malarial parasite, the microbe of relapsing fever and the plasmodium of malaria having a striking morphological resemblance.

Under the head of masked intermittent fever, some authors report the various manifestations. The term is calculated to mislead the enquirer, for fever does not always attend masked intermittent. As often as otherwise, perhaps, the thermometer indicates no rise in temperature, or if any, so slight and continued that we must conclude the malarial poison has made no febrile impression. Intermittent periodicity is, perhaps, always marked, but not fever. Modern authors upon the practice of medicine limit their review upon malaria to the different types of malarial fever; but he who restricts its manifestations to the specific forms of fever, is not prepared to practice medicine in malarial districts. Griesinger includes among masked intermittents, the most diverse nervous affections, to-wit: Convulsions, localized or general, choræic or epileptiform; hysteria, amblyopia, temporary paralysis of some of the members, and even symptoms of a different order, as a partial or general œdema. Colin, it is true, protests against this wholesale appropriation of so many diverse diseases to malarial influence, and calls attention to the fact that quinine has a beneficial effect upon nervous disease of non-malarial origin. Griesinger did not intend to assert that these diseases were not generally non-malarial, but that malarial manifestations often simulated them.

The diseases enumerated by Griesinger, when not malarial, many of them, are benefited by quinine, but this remedy never aborts them. I hold that any disease in which quinine is an abortifacient, is malarial. Solibert mentions among the list of masked intermittents: Motor malarial paralysis, spasmodic affections, convulsions, contractions, muscular atrophy, hemiplegia, aphasia, transient paralysis. Harlan mentions bitemporal hemianopsia. Lewis tells of malaria among children masking as bronchitis. A case is reported, by whom I do not now remember, of a woman, of sixty-four years of age, suddenly seized with paralysis of the lower extremities and sphincters, sensibility unchanged, consciousness clear, temperature normal (mark that!), pulse eighty, small and empty, no pain in the spinal region. After twelve hours, these symptoms left her, to return the next day—complete intermissions each day—until quinine was given, when all symptoms left suddenly. Intermittent fever is often manifested in local symptoms, restricted, perhaps, to some member of the body, arm or leg. I treated a case in 1865, in which the ear, at 10 a. m., would redden and burn, and severe pain would set up, disappearing at about 5 p. m., to return the next day. The general temperature remained normal. Quinine relieved this case immediately. Similar cases are reported of local intermittents of arm and leg. Some years ago I was very much interested in this subject, and kept notes of phenomenal cases, which I still have, but which are not now available. I regret not having them convenient, for they are interesting. I recall a case of epilepsy in a young negro girl, aged 16 years, occurring at intervals of four weeks, at catamenial periods, recurring each day for three successive days, and stopping without remedies, to return at the succeeding period. The menstrual flow was full and without pain. I diagnosed hysterical epilepsy, and treated her accordingly, without relief. It dawned upon me that there was a marked intermittent character to the symptoms, and quinine was administered with radical relief. I have seen cases of dismenorrhœa, coming on suddenly and without apparent cause, continuing at each menstrual period, resisting all other treatment, and yield immediately to qninine. I have seen cases of periodic hæmaturia, not so-called black jaundice, but simple intermitting kidney hemorrhage, without fever, yield quickly to quinine. Again, I have seen periodical intermitting diarrhœa, resisting all other treatment, yield to quinine. There are many other phenomenal manifestations which I might name, many of which I have seen, masking in the form of cutaneous diseases, but I am warned that I have trespassed upon your patience long enough. The sequelæ of malarial infection do not come within the scope of this paper; a very interesting paper might be written upon these alone. I cannot, however, refrain from mentioning, incidentally, that several years ago I had a case of cirrhosis of the liver which I pronounced of malarial origin. I was laughed at by the consulting physician, who charged me with being crazy upon this subject. I searched over medical literature at the time, upon the subject, and could find nothing sustaining me; but, like the fellow who swore the horse was eighteen feet high, I stuck to it. In the Annual of the Universal Medical Science of 1889, I read that Dr. Lancereaux, of Paris, had delivered a series of lectures in one of the hospitals upon the subject of cirrhosis of the liver from malarial infection. I immediately called the attention of my medical friend to the report.

During the period in which I was engaged in special observation upon this subject, I observed that malarial chill seldom made its first appearance in the night, viz: between 10 p. m. and 3 a. m.; when it does, it is a rare exception. When called to a case of chill, and subsequent fever, between these hours, I feel confident that it is not malarial, but the precursor of, perhaps, a more formidable malady. The observations of several years have not changed this opinion.

Is malarial fever contagious? Some of you may laugh at the question, so confident are you that it is not. I do not believe it is, still medical men of high authority upon malaria, hold that it is contagious. Sternberg, to whom I am indebted for several facts mentioned in this paper, reports cases which seemingly point that way. Bessingneits reluctantly, he says, was convinced that it could be communicated from one person to another. He reports twelve examples, all healthy and not otherwise exposed, who were seized with ague, after sleeping with one during the sweating stage of the paroxysm.

It is important that an early diagnosis of these phenomenal cases should be made, for early in the acute stage is the favorable time for a radical cure. Delay might be dangerous; an organic lesion might be the result, or the case might become chronic, when quinine will not always act as an abortifacient.

What test have we of malarial disease? At the time of the cases reported by me, I had no infallible test, as we have now, though I regard quinine, if an abortifacient, wellnigh infallible. The history, the tongue sometimes, when full, filling the mouth and indented, with the quinine test, were the landmarks for my diagnosis. When the two former were absent, the thereapeutical effect of quinine was my test; but now we have a test which all of you will recognize—the presence of the hæmatozoon, or plasmodium malariæ, in the blood. This is as yet the only practical benefit of our advanced knowledge of the etiology of the disease. They are always present in malarial diseases. A case in point, reported by Brandt, a person was seized with coma who had received an injury to the head. Every preparation had been made for a surgical operation. The surgeon determined to make a microscopic examination of the blood before operating. The plasmodium malariæ was found; the operation was abandoned, and quinine administered. The patient rapidly recovered and a life was saved. Surgical interference might have resulted in death, and under no circumstances could have been of any benefit.

---

DISCUSSION OF DR. CLOPTON'S PAPER.

*Dr. Allen J. Smith*, objecting to the implied views of the writer that the malarial parasite was of bacterial nature, called the attention of the members to the fact that the organism discovered in the blood of malarial patients by Laveran is not of vegetable but of animal nature. He reviewed the former teachings as to the bacterial origin of paludism, and related the history of the discovery of the hæmatozoon of Laveran, and the gradual development and popularization of the present views of the subject.

Having called the attention of the members to a microscope with one of the hæmatozoa in the field, the speaker then demonstrated in detail the various forms assumed by the organism in question, and the probable succession of events in the life history of the parasite as far as is known at present, employing a number of lecture diagrams to illustrate his remarks, embodying detailed instruction as to the technique necessary for its observation and for its preservation and staining. He stated his belief that this organism is the unfailing concomitant of malarial fever, and probably its cause, basing this belief upon the uniformity of its discovery in the blood of malarial cases if properly sought for, and its uniform failure in the blood of patients affected by other forms of disease or in the normal blood, by the uniform success of observers the world over, many of them far from the original place of the discovery and in no way influenced by personal views of the discoverer, by the frequent discovery of this organism in masked forms of malaria, perhaps unsuspected before, but yielding readily to quinine. He agreed entirely with Dr. Clopton that apparently very dissimilar maladies are often due to this cause, narrating within his own experience, a case of coma, and one of chorea, recognized in their true light only upon the discovery of the hæmatozoon of malaria, both yielding promptly and easily to the malarial specific, quinine. He had also met with cases of malaria which had been viewed as cases of Bright's disease in advanced stages at first, but whose subsequent course, in view particularly of the demonstration of the malarial organisms in their blood, had left no doubt as to their paludic nature. Commenting upon Dr. Clopton's scriptural adaption to the Italian, Golgi, the speaker admitted his concurrence, and said that in his opinion Golgi's differentiation of the parasites into special groups, as related to the varied common manifestations, is not warranted by our present knowledge of the subject; and urged the credibility of Laveran's view that the parasite bearing his name is multiform, but single. In answer to Dr. Clopton's statement, that in spite of the discovery of Laveran, we are no nearer to a satisfactory explanation of the manifestations of malaria, the speaker reminded the writer that some of its nervous phenomena might be explained by the well-known embolism of the vessels in the central nervous system by these hæmatozoa.

*Professor Keiller:* Dr. Keiller thanked both Dr. Clopton and Dr. Smith for the valuable exposition they had given of the cause and manifestations of malarial fever. Having come from a country (Scotland) where malarial fever is unknown, and where imported cases are rarely met with, he felt that his knowledge of the subject was such as to make the exposition he had just heard peculiarly valuable to him. He thought, however, that from the multiform manifestations credited to the malarialial organisms, his difficulty in the future would be not in deciding what was, but what was not, malarial fever. He also expressed the opinion that extreme care should be exercised diagnosing a case as malarial without microscopic proof; otherwise disastrous mistakes would certainly and frequently result.

*Professor Thompson:* Dr. Thompson referred to the frequency and persistency with which the internes at John Sealy Hospital would tentatively drop the suggestion that such and such manifestations might be due to malaria; and he emphasized the view that this tendency to regard various maladies as due to malarial poisoning, when no microscopic proof was forthcoming, as distinctively savoring of charlatanism. He held that no case should be diagnosed as malarial unless the microscope revealed the presence of Laveran's hæmatozoon.

*Professor Clopton:* Dr. Clopton said, that notwithstanding the discovery of the hæmatozoon of malaria, we are no nearer to a satisfactory explanation of the various forms of maralial fever, or of how its manifestations are really produced.

